# Pharmacogenomics of cardiovascular drugs

**DOI:** 10.1186/1755-8166-7-S1-I53

**Published:** 2014-01-21

**Authors:** C Adithan

**Affiliations:** 1 Department of Clinical Pharmacology, JIPMER, Pondicherry, India

## 

Cardiovascular diseases account for the second largest number of non-communicable disease after mental illnesses. Coronary heart diseases, hypertension, atherosclerosis, congestive heart failure, arrhythmias are important cardiovascular diseases. Beta blockers, statins, antiplatelet drugs, anticoagulants, drugs modifying renin angiotensin systems and many others are being used for treating these ailments. Still, satisfactory treatment of these diseases is still elusive. Drug response in influenced by many environmental factors besides host factor. Besides the above, genetic factor is also important in modifying response to cardiovascular drugs. Now there is reasonable amount of evidence exists that Indian population is genetically distinct from other major ethnic groups. The allele and genotype frequencies of genes encoding important drug metabolizing enzymes, drug transporters and receptors of Indian population are different from Caucasians and Orientals. Studies done in our laboratory and other Indian laboratories suggested that pharmacogenomics of clopidogrel, warfarin, acenocoumarol, beta blockers etc. may have clinical significance in treating cardiovascular diseases of Indian population. There is a need for multi-institutional and multi-disciplinary approach for large scale implementation of pharmacogenomics and personalized medicines in cardiovascular diseases.

